# Photovoltaic Characteristics of GaSe/MoSe_2_ Heterojunction Devices

**DOI:** 10.1186/s11671-021-03630-y

**Published:** 2021-11-29

**Authors:** Ryousuke Ishikawa, Pil Ju Ko, Ryoutaro Anzo, Chang Lim Woo, Gilgu Oh, Nozomu Tsuboi

**Affiliations:** 1 Advanced Research Laboratories, Tokyo City University, Tokyo, Japan; 2 Department of Electrical Engineering, Chosun University, Gwangju, Republic of Korea; 3 Department of Materials Science and Technology, University of Niigata, Niigata, Japan

**Keywords:** 2D materials, Heterojunction, Solar cell, GaSe, MoSe_2_

## Abstract

The two-dimensional materials have the thickness of an atomic layer level and are expected as alternative materials for future electronics and optoelectronics due to their specific properties. Especially recently, transition metal monochalcogenides and dichalcogenides have attracted attention. Since these materials have a band gap unlike graphene and exhibit a semiconductor property even in a single layer, application to a new flexible optoelectronics is expected. In this study, the photovoltaic characteristics of a GaSe/MoSe_2_ heterojunction device using two-dimensional semiconductors, p-type GaSe and n-type MoSe_2_, were investigated. The heterojunction device was prepared by transferring GaSe and MoSe_2_ onto the substrate which the titanium electrodes were fabricated through a mechanical peeling method. The current–voltage characteristics of the GaSe/MoSe_2_ heterojunction device were measured in a dark condition and under light irradiation using a solar simulator. The irradiation light intensity was changed from 0.5 to 1.5 sun. It was found that when the illuminance was increased in this illuminance range, both the short-circuit current and the open-circuit voltage increased. The open-circuit voltage and the energy conversion efficiency were 0.41 V and 0.46% under 1.5 sun condition, respectively.

## Introduction

Two-dimensional (2D) materials have been found to have various unique characteristics that are not an extension of conventional materials science [1–5]. In particular, they are attracting attention as optoelectronic materials owing to the notable physical properties such as their strong optical absorption in the solar spectrum region [6], high internal radiative efficiencies [7], and tunable band gaps for both single- and multi-junction solar cells [8]. Some solar cells are made of 2D materials by forming in-plane and out-of-plane heterojunctions. The former is characterized in that a very clean heterojunction interface can be formed by continuously growing different types of 2D materials [9, 10]. On the other hand, in the latter case, since the heterojunction area can be increased, and tandem solar cells can be fabricated by stacking several junctions, the solar cell characteristics of the GaSe/MoSe_2_ vertical heterojunction device were evaluated in this study.

Gallium selenide has long been expected as an optical material for photodetectors and nonlinear optics, but its practical application has been promoted only in limited situations due to the difficulty of synthesizing single crystals [11–13]. However, due to recent advances in two-dimensional materials science, this layered optical material has been attracting attention again [14–21]. MoSe_2_ is a typical transition metal dichalcogenide, the Mo ion in these compounds is surrounded by six Se^2−^ ions. The coordination geometry of the Mo is found as octahedral and trigonal prismatic. Monolayer MoSe_2_ exhibits semiconducting properties with a direct bandgap of about 1.6 eV and has relatively high carrier mobility on the order of hundreds [22]. Therefore, MoSe_2_ is attracting attention not only as optoelectronics but also as an active region material for transistors [23, 24].

These 2D material heterojunctions have high potential as solar cell materials due to the properties already described that very high theoretical conversion efficiencies for single- and tandem-junctions have been demonstrated thanks to high external radiative efficiency [8], but conversion efficiencies reported so far due to inadequate material and interface quality and device design [25–27]. Furthermore, there are still many unclear points about the device physics in the out-of-plane heterostructure of 2D materials, especially the carrier separation process, which is important in solar cells.

In this paper, the current-voltage characteristics of the GaSe/MoSe_2_ heterojunction device fabricated through a mechanical peeling method were measured in a dark condition and under light irradiation using a solar simulator. The irradiation light intensity was changed from 0.5 to 1.5 sun. It was found that when the illuminance was increased in this illuminance range, both the short-circuit current and the open-circuit voltage increased. The open-circuit voltage and the energy conversion efficiency were 0.41 V and 0.46% under 1.5 sun condition, respectively.

## Methods

We fabricated four-terminal devices using 50 nm of thickness titanium (Ti) electrodes deposited by electron-beam evaporation on p-type silicon substrates covered with 300 nm of thermally oxidized silicon dioxide (SiO_2_). We transferred flakes of natural GaSe and MoSe_2_ (HQ graphene) onto the Ti electrodes sequentially using polydimethylsiloxane (PDMS, Dow Toray) by mechanical exfoliation as described in previous report [23]. Finally, the Ti/GaSe/MoSe_2_ heterojunction device was annealed at 400 °C under nitrogen gas atmosphere for two hours. The transmittance and reflectance spectra in a few ten micro meters square areas were obtained using transferred flakes onto glass substrates by a micro-UV–Vis spectrometer with a wide-band cassegrain objectives lens (JASCO MSV-5300). The thickness of each sample flakes was determined from line profile of atomic force microscopy (AFM) images (HITACHI Nano Navi Real). The micro-PL and Raman measurements were conducted with a continuous wave excitation laser emitting at 532 nm coupled to a 100× microscope objective at 25 °C. The excitation light intensities for Raman and PL measurements were 1.5 and 0.3 mW, respectively. The solar cell performance was measured at a sample temperature of 25 °C using a solar simulator with a variable intensity between 0.5 sun and 1.5 sun. The spectral response was evaluated by combining a monochromatic light source and a pico-ammeter. From the optical microscopic image, the heterojunction region was determined as the active area of solar cells.

## Results and Discussion

**Fig. 1:**
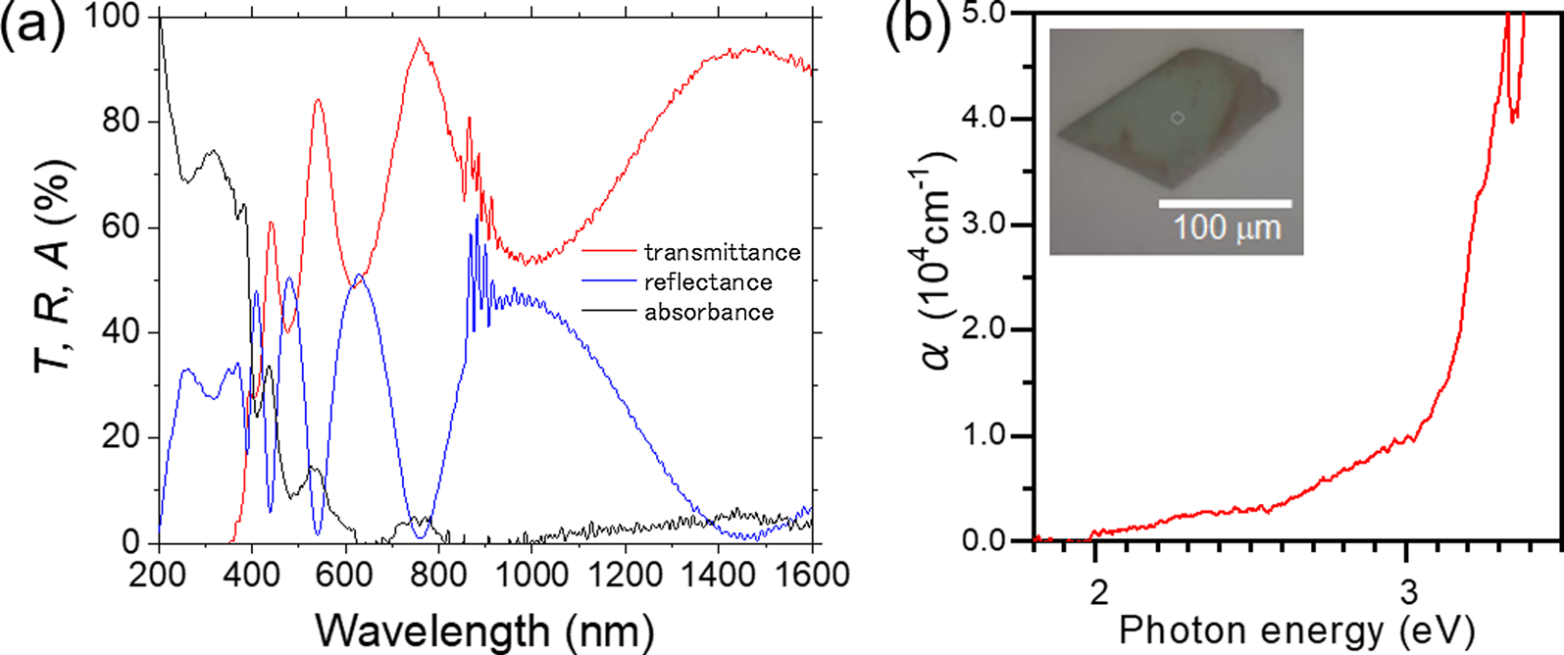
**a** Transmittance, reflectance, absorbance spectra and **b** absorption coefficient of GaSe flake. Inset: optical microscope image of GaSe flake

Figure 1a shows the transmittance (*T*) and reflectance (*R*) spectra of GaSe flake on glass substrates. The solid red and blue lines show the measured transmittance and reflectance spectra in the range of 200–1600 nm, respectively. The absorbance spectrum (*A*) represented by a solid black line was calculated by following relation;

1 $$A = 1 - T - R$$

**Fig. 2:**
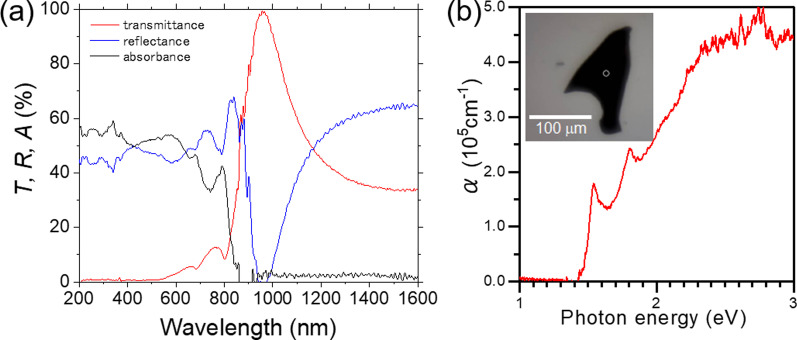
**a** Transmittance, reflectance, absorbance spectra and **b** absorption coefficient of MoSe_2_ flake. Inset: optical microscope image of MoSe_2_ flake

The absorption coefficient was calculated by following equation as shown in Fig. 1b.

2 $$\alpha = \frac{{\ln \left( {1 - R} \right) - \ln T}}{d}$$

where *d* is thickness of sample, which was estimated to be 638 ± 29 nm by AFM measurement. The absorption coefficient of GaSe gradually increased from around 2 eV corresponding to the bandgap. Since the valence band maximum exists at Γ-point, and the bottom of the conduction band at Γ-point is only a few tens meV above the conduction band minimum at M-point, GaSe is considered a quasi-direct bandgap [12]. Direct excitons are also known to be at the Γ-point of energy very close to the direct and indirect interband transitions [12, 19]. Inset of Fig. 1b shows the optical microscope (OM) image of GaSe flake for measurement. The centered circle in OM image indicates measuring area. On the other hand, Fig. 2 shows the optical properties of MoSe_2_ flake with the thickness of 99 ± 3 nm transferred on glass substrates. The absorption coefficient of MoSe_2_ exhibited more than an order of magnitude higher than that of GaSe. The sharp increase from 1.5 eV and two exciton-oriented peaks were compatible to previous reports [28, 29].

**Fig. 3:**
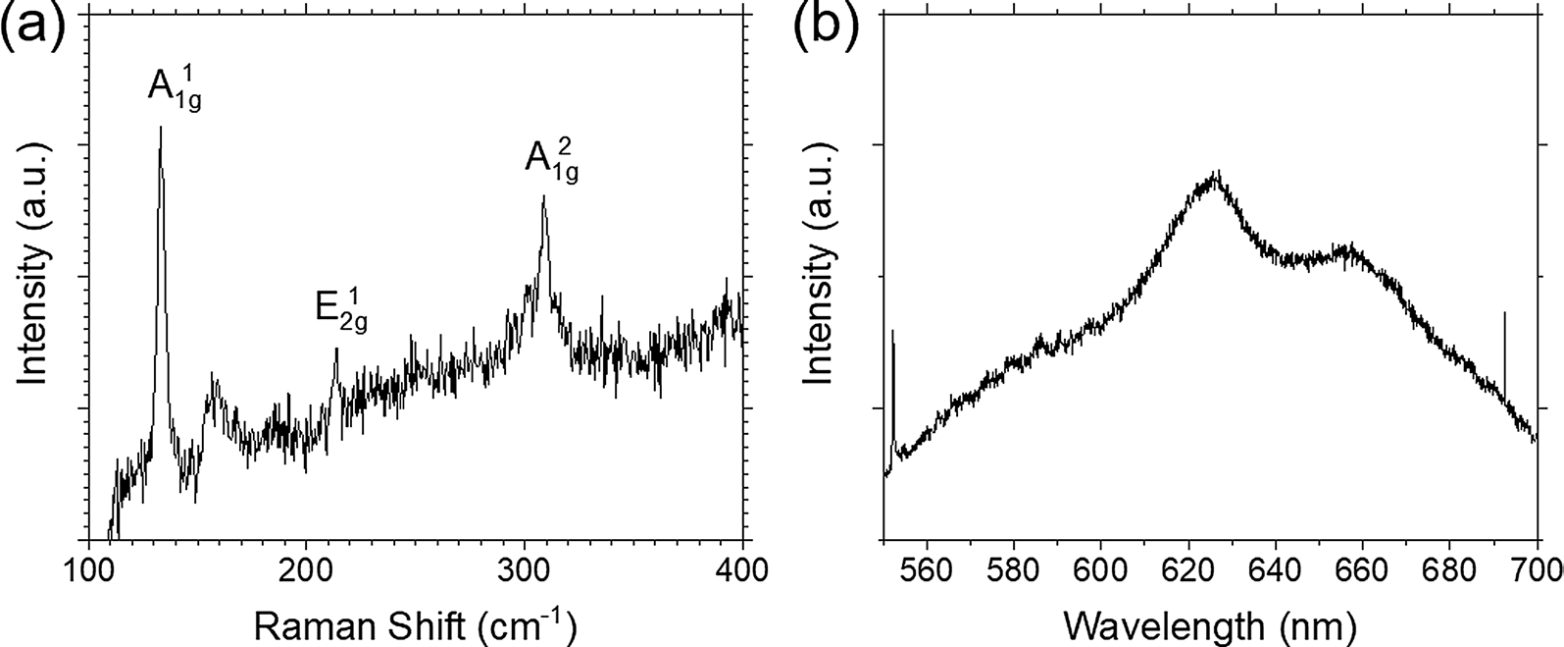
**a** Raman and **b** PL spectra of GaSe flake

**Fig. 4:**
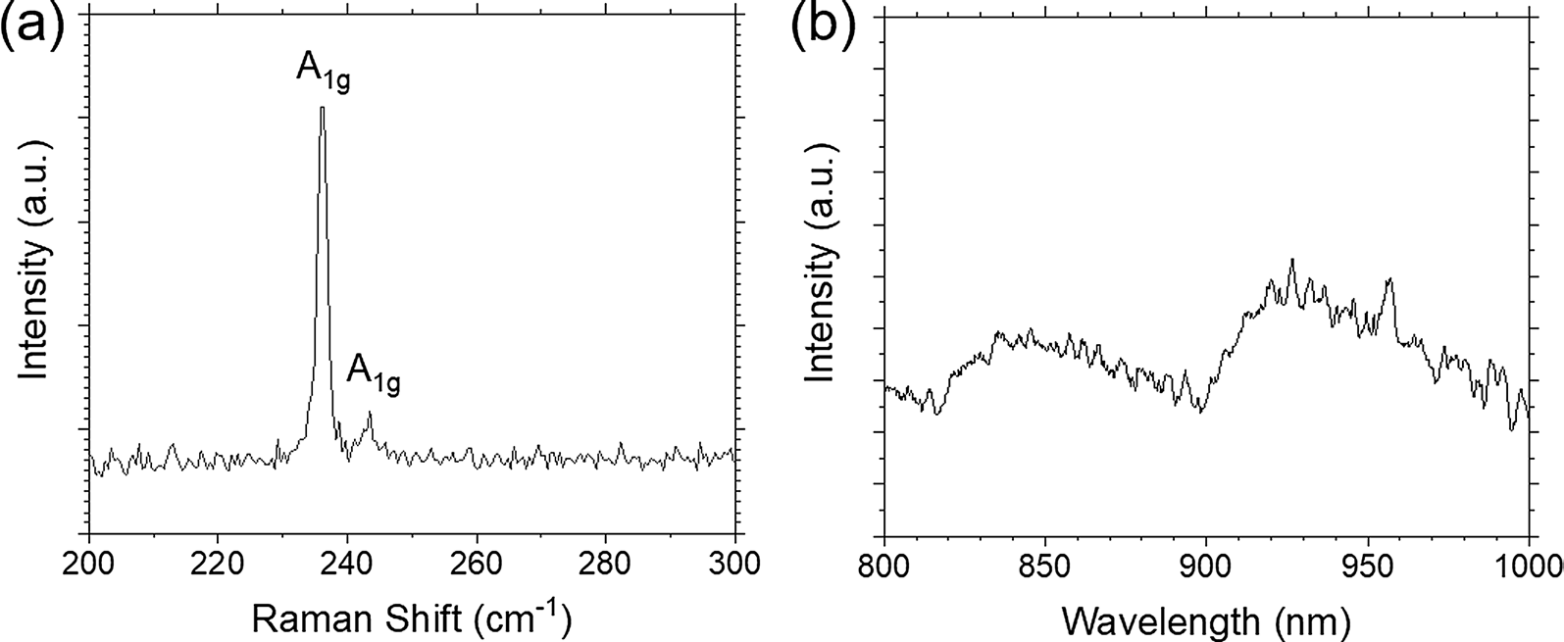
**a** Raman and **b** PL spectra of MoSe_2_ flake

Next, the crystallinity and further optical properties of these two-dimensional materials were investigated by Raman and PL. Raman and PL spectra were measured using fabricated GaSe/MoSe_2_ heterojunction devices. The Raman peaks at 133, 214, and 309 cm^−1^ were observed as shown in Fig. 3a. The Raman peaks at 133 and 309 cm^−1^ indicate the planar vibrational modes of A^1^_1g_ (133 cm^−1^) and A^2^_1g_ (309 cm^−1^), respectively. The other peak at 214 cm^−1^ comes from the vibration of selenides in the out-of-plane mode so called E^1^_2g_ [15, 17]. These clear crystalline vibrations indicate high crystallinity of transferred GaSe flakes. Figure 3b shows the PL spectrum obtained from GaSe flakes on Si substrate at 25 °C. The PL peaks arounds 626 and 655 nm corresponding to the direct and the indirect bandgaps, respectively. The indirect bandgap sets only 25 meV lower than the direct bandgap in GaSe [18, 19]. The Raman spectra of MoSe_2_ transferred on Si substrates indicated two obvious peaks at around 236 and 243 cm^−1^, which are corresponding to A_1g_ mode as shown in Fig. 4a. The Raman and luminescence spectra (Fig. 4b) indicate high quality of transferred MoSe_2_ flakes on Si substrates.

**Fig. 5:**
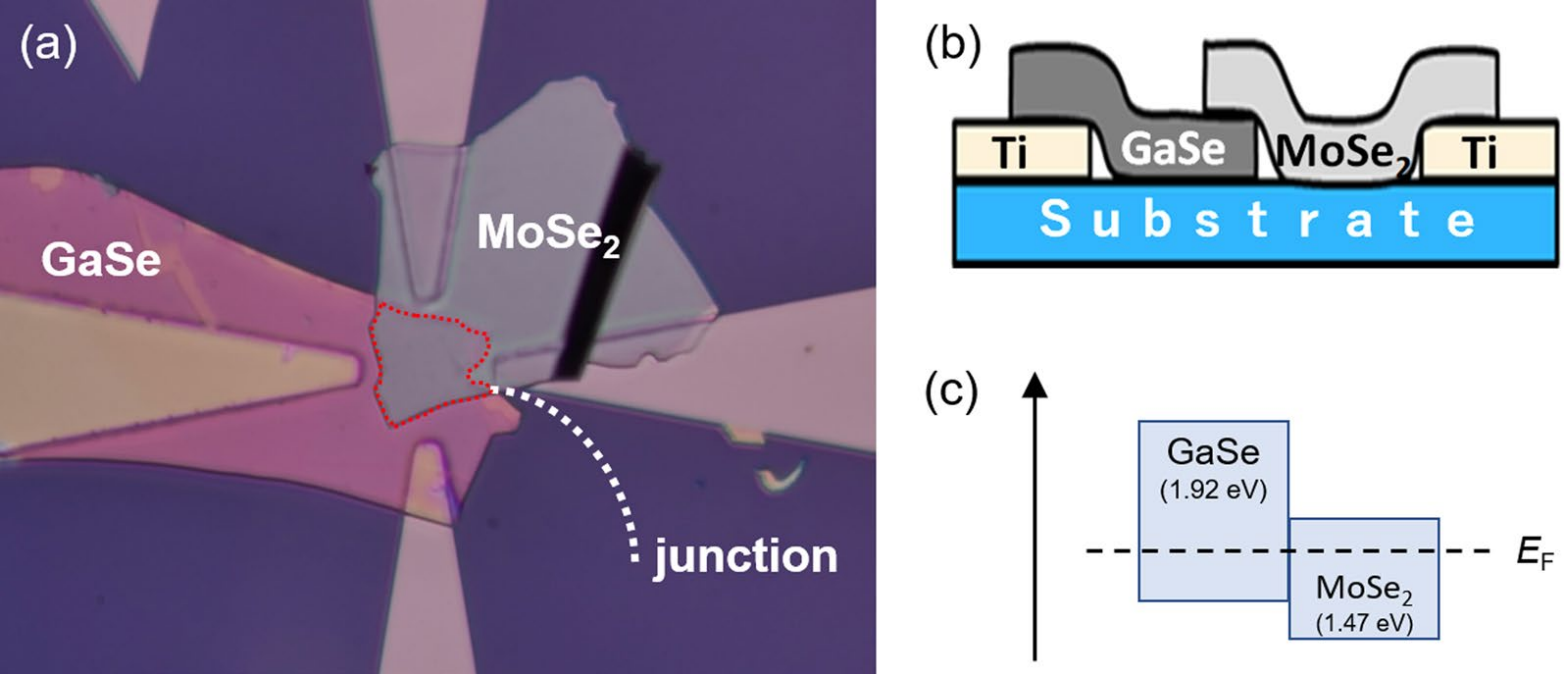
**a** Optical microscopic image, **b** schematic image, and **c** band diagram of the fabricated GaSe/MoSe_2_ heterojunction device

Figure 5a shows the optical microscopic image of the fabricated GaSe/MoSe_2_ heterojunction device contacted with Ti electrodes. The GaSe flake is contacted with left and bottom electrodes, and the MoSe_2_ flake is contacted with right and top electrodes, respectively. The heterojunction region defined as the active area of solar cells was estimated to be 490 μm^2^ from this image. The solar cell performance was measured using bottom and top electrodes under simulated sunlight. The thickness of these GaSe and MoSe_2_ flakes were estimated to be 118 and 79 nm by AFM measurement, respectively. Both of these film thicknesses correspond to 120–130 layers. Schematic image and band diagram of GaSe/MoSe_2_ heterojunction device were illustrated in Fig. 5b, c, respectively.

**Fig. 6:**
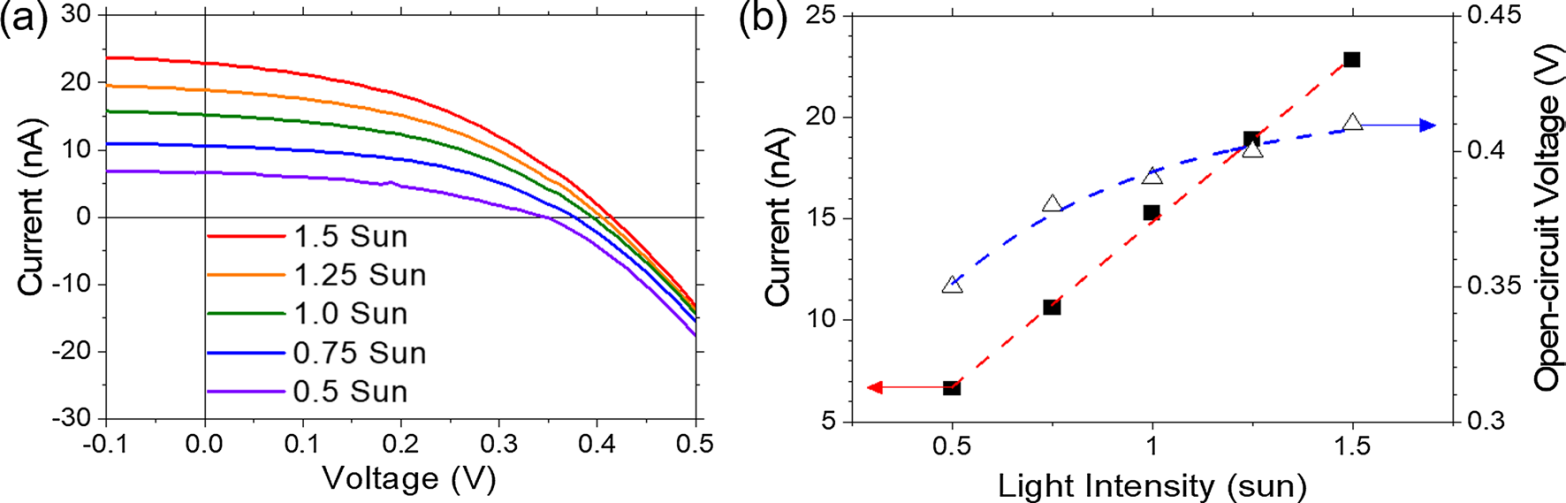
**a** *I*–*V* characteristics and **b** light irradiation intensity dependence of GaSe/MoSe_2_ heterojunction solar cell performance

The current-voltage characteristics of the fabricated GaSe/MoSe_2_ heterojunction device under 0.5–1.5 sun light condition are shown in Fig. 6a. It is clear that this heterojunction device exhibits rectification and photovoltaic effect, and it can also be seen that the *I*–*V* curve changes depending on the light irradiation intensity from Fig. 6a. Figure 6b shows a summary of the light irradiation intensity dependence of the short-circuit current (*I*_sc_) and the open-circuit voltage (*V*_oc_). *I*_sc_ increases linearly with light irradiation intensity in this range. On the other hand, it can be seen that *V*_oc_ increases logarithmically with respect to the light irradiation intensity. Since the following relational expression holds for an ideal diode, the ideal factor was estimated to be 1.11 by fitting.

3 $$V_{{{\text{oc}}}} = \frac{{nk_{{\text{B}}} T}}{q}\ln \left( {\frac{{I_{{\text{L}}} }}{{I_{{{\text{dark}}}} }} + 1} \right)$$

where *n* is the ideality factor, *k*_B_ is the Boltzmann constant, *T* is the temperature of the device, *q* is the fundamental unit of charge, so that $\frac{{k_{{\text{B}}} T}}{q} \approx$ 0.0258 V at room temperature. The *I*_L_ and *I*_dark_ are photo- and dark-current, respectively. An ideal factor closes to 1 indicates that this GaSe/MoSe_2_ structure forms an ideal heterojunction in which an internal electric field sufficient to dissociate excitons is present. The short-circuit current density (*J*_sc_) was calculated to be 3.11 mA/cm^2^ from active area defined by optical image. The fill factor (*FF*) and conversion efficiency (*η*) were estimated to be 0.44 and 0.54% under 1 sun condition, respectively. Since the *FF* decreased due to the influence of the series resistance when irradiating for 1 sun or more, the *η* was almost the same as when irradiating for 1 sun, although the *J*_sc_ and the *V*_oc_.increased. In order to improve *FF*, it is necessary to improve the device configuration such as shortening the distance to the electrode.

**Fig. 7:**
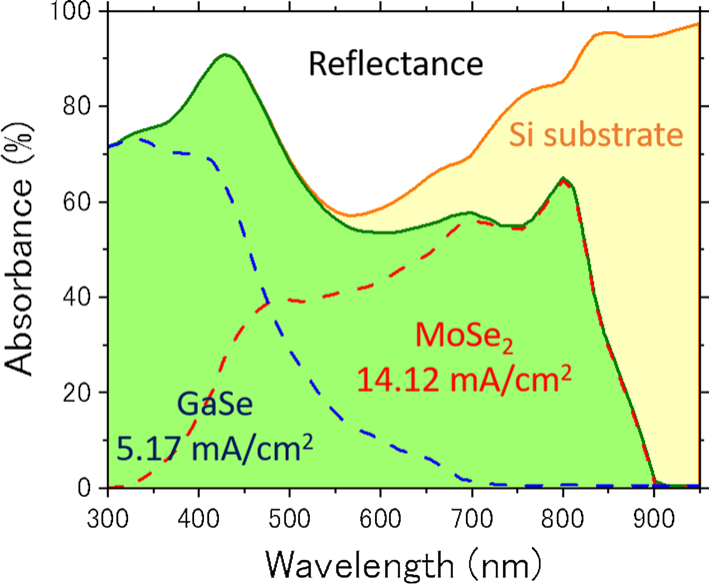
The simulated absorbance spectra of GaSe/MoSe_2_ heterojunction

Next, we estimated the external quantum efficiency of the GaSe/MoSe_2_ heterojunction by using an optical simulator (e-ARC) [29]. Calculations were made with a completely flat structure in which GaSe and MoSe_2_ with the same film thickness as the fabricated device were laminated on a flat Si substrate. The optical constants of GaSe and MoSe_2_ were referred to the reported values [30, 31]. The carrier loss induced by recombination at material interface and bulk regions are fully incorporated. The simulated absorbance spectra are shown in Fig. 7. The green-colored region shows the absorption region of the GaSe/MoSe_2_ heterojunction, which is the sum of the absorption of GaSe indicated by the blue dashed line and the absorption of MoSe_2_ indicated by the red dashed line. The yellow region is transmitted and absorbed by the Si substrate, and the other regions show reflection components. The maximum *J*_sc_ over the wavelength range of 300–950 nm was estimated to be 19.29 mA/cm^2^ if the generated photocarriers could be completely collected from fabricated device. Our simulation results predicted that the *J*_sc_ would increase, and 23 mA/cm^2^ could be obtained when the GaSe film thickness was about 60 nm. The large dissociation between the calculated current value and the experimental value may be due to insufficient built-in potential in the fabricated device. If this hypothesis is correct, optimizing the film thickness of the absorbent layer and optimizing the work function of the contact material could significantly improve the *J*_sc_. Furthermore, since this simulation result shows that the reflection component is also large, it can be said that the light confinement effect on the incident surface side and the back surface side of the GaSe/MoSe_2_ heterojunction solar cell is also an important issue in the future. Surface plasmon technology is considered to be very effective for light confinement in two-dimensional material-based solar cells [32].

## Conclusions

In conclusion, we fabricated the GaSe/MoSe_2_ heterojunction devices through a mechanical peeling method and analyzed the photovoltaic performance. The absorption coefficient obtained from transmittance and reflectance spectra of MoSe_2_ exhibited more than an order of magnitude higher than that of GaSe. The Raman and luminescence spectra of GaSe and MoSe_2_ indicated that high crystallinity maintained after device fabrication. Both the short-circuit current and the open-circuit voltage increased when the light intensity is increased from 0.5 to 1.5 sun. The open-circuit voltage and the energy conversion efficiency were 0.41 V and 0.46% under 1.5 sun condition, respectively. The maximum *J*_sc_ over the wavelength range of 300–950 nm was estimated to be 19.29 mA/cm^2^ if the generated photocarriers could be completely collected from fabricated device from optical simulation study. The optimizing the film thickness of the absorbent layer and optimizing the work function of the contact material could significantly improve the *J*_sc_. Furthermore, the light confinement effect on the incident surface side and the back surface side of the GaSe/MoSe_2_ heterojunction solar cell is also an important issue in the future.

## Data Availability

The datasets supporting the conclusions of this article are included within the article.

## Abbreviations

2D materials: Two-dimensional materials
AFM: Atomic force microscopy
OM: Optical microscope
*I*_sc_: Short-circuit current
*V*_oc_: Open-circuit voltage
*J*_sc_: Short-circuit current density
*FF*: Fill factor
